# Patient Experiences of Structured Heart Failure Programmes

**DOI:** 10.1155/2010/157939

**Published:** 2011-02-07

**Authors:** Nuala E. Tully, Karen M. Morgan, Helen M. Burke, Hannah M. McGee

**Affiliations:** Division of Population Health Sciences, Department of Psychology, Royal College of Surgeons in Ireland, 123 St. Stephen's Green, Dublin 2, Ireland

## Abstract

*Objectives*. Patient experiences of structured heart failure rehabilitation and their views on the important components of heart failure services were examined. *Methods*. Focus groups were conducted with fifteen participants (men, *n* = 12) attending one of two heart failure rehabilitation programmes. Sessions were guided by a semistructured interview schedule covering participants' experiences of the programme, maintenance, and GP role. Focus group transcripts were analysed qualitatively. *Results*. Participants indicated that rehabilitation programmes substantially met their needs. Supervised exercise sessions increased confidence to resume physical activity, while peer-group interaction and supportive medical staff improved morale. However, once the programme ended, some participants' self-care motivation lapsed, especially maintenance of an exercise routine. Patients doubted their GPs' ability to help them manage their condition. *Conclusion*. Structured rehabilitation programmes are effective in enabling patients to develop lifestyle skills to live with heart failure. However, postrehabilitation maintenance interventions are necessary to sustain patients' confidence in disease self-management.

## 1. Introduction

The combined effects of an ageing population and advances in treatments for acute myocardial infarction have resulted in heart failure becoming what is known as a silent epidemic in industrialised nations [1]. Heart failure is associated with severe symptom burden, functional limitation, and premature death [2]. Treatment regimens are complex, and there are many barriers to adherence. Unsurprisingly, patients often report poor quality of life, high levels of psychological distress, and difficulties adhering to treatment regimens. 

Heart failure presents a diagnostic and management challenge as most heart failure patients are older, have multiple comorbidities, and may be on multiple treatment regimens. In addition, the signs and symptoms of heart failure are nonspecific, which can be a barrier to early diagnosis, essential in preventing disease progression and reducing hospitalisation. A further complication is that many patients are never told they have heart failure due to clinician reluctance to use the term [3]. 

Heart failure places significant burden on primary and secondary care services. The direct medical costs of heart failure treatment constitute from 1% to 2% of total health-care expenditures in developed countries [4]. This proportion exceeds that used by HIV or cancer. A significant part of this cost is due to high hospital readmission rates. While hospitalisations for most other cardiovascular conditions have remained static or decreased over the past number of decades, hospitalisations for heart failure have increased threefold [5]. 

Treatment requires a complex combination of pharmacological and lifestyle interventions. Changes in lifestyle and symptom monitoring are important complementary strategies to pharmacological treatment. Lifestyle changes include the reduction of salt intake, fluid restriction, alcohol restriction, and modifying physical activity. Symptom monitoring is essential to detect deterioration in the patient's condition, for example increased shortness of breath, weight gain, and oedema (fluid retention). It includes activities such as daily weighing to monitor oedema. Taken together, these treatment regimens can be very demanding on the patient. Structured cardiac rehabilitation plays an essential role in helping patients adopt new lifestyle practices following their diagnosis of heart failure. Comprehensive programmes combining exercise and education have been found to improve physical status and psychosocial well-being, as well as reducing hospital readmissions [6]. Developing an in-depth understanding of the disease in patients is a fundamental aspect of therapy and essential for adherence to self-management practices [7].

Neglected in the literature around the subject of heart failure is a description of the patient's perspective on rehabilitation programmes. The purpose of this study was to explore patients' experiences of these programmes, including their views on the important components of heart failure services, and to examine their quality of life after rehabilitation.

## 2. Methods

While much quantitative research seeks to draw conclusions that can be generalised, qualitative research aims to provide an insight into the meaning of the everyday experiences of the participants. To explore the experiences of people living with heart failure, focus groups [8] were conducted with fifteen participants attending one of two group heart failure rehabilitation programmes. Each programme was based in an acute general hospital (one urban, one rural). Fifteen participants took part, seven from the rural hospital and eight from the urban hospital. In the rural hospital all participants were male and in the urban hospital five of the eight participants were also male. Other demographic data was not collected as the researchers wanted participants to talk openly and honestly about their experiences of the heart failure service. The heart failure services in both hospitals were limited in size and other demographic data was not gathered in order to reassure participants about confidentiality and anonymity. All participants had been diagnosed for at least one year. In each hospital patients that had completed heart failure rehabilitation were approached by the hospital's heart failure rehabilitation coordinator to participate in the study. Two focus groups were carried out, one in each hospital. One researcher facilitated both focus groups and another researcher co-facilitated the focus group held in the rural hospital. Sessions were guided by a semistructured interview schedule, with questions covering various aspects of the patient's illness experiences including:

- experience of the cardiac rehabilitation programme,
- beliefs about their diagnosis of heart failure,
- maintenance and self-care for their condition, and
- the role of the GP in caring for patients with heart failure.

The order of questions varied according to participants' responses and followup questions were used to gain further detail on the topics. The discussions were flexible and allowed participants to speak freely of their experiences living with heart failure and to raise issues important to their lives. The focus groups took place in a quiet room within both hospitals, and no hospital staff were present for the duration of the discussion. Each session lasted approximately one hour and was taped with the participants' permission. 

The focus groups were transcribed verbatim and the resulting data was qualitatively analysed using content analysis [9]. This generated a more concise set of emergent codes. These codes were further examined, and this led the researcher to identify the major themes that emerged from the data. One researcher carried out all of the analysis and interpretation of the transcripts. The study protocol was approved by the Research Ethics Committee of the Royal College of Surgeons in Ireland.

## 3. Results

Themes identified from the focus group discussions included improvements in confidence and morale, difficulties in maintaining self-care, attitudes to GP services, frustrations with quality of care in other healthcare settings, coping with the medication regime, and attitudes to the term “heart failure”.

## 4. Experience of Programmes

The programmes bolstered the confidence of participants by providing a benchmark of their health status. Many started rehabilitation uncertain of the limits within which they could safely be physically active and fearful of the consequences if they exceeded this unknown threshold. Participants repeatedly mentioned how the monitoring of their heart rate by the medical staff during the exercise sessions provided a sense of security.

*I thought that maybe this thing will creep up behind me some day, but having done the course here, you'd be on a monitor and you saw your reactions to, you knew what you could do and what you couldn't do …. That settled me down and I was quite happy then. (Centre A, R4).*

*It was a great reassurance for me, anyway, personally, a great confidence booster. And I'm not afraid to do things now. And I was certainly afraid before I took the course. (Centre A, R1).*

The programme of exercise was a crucial kick-start to becoming functional and mobile once more.

*When I came here first I could hardly lift a cup of water and, sure, I could pick two big buckets now and go where I like with them. I'd be very fit now, I couldn't be much fitter. (Centre A, R7).*

The patient-centred approach and the focus on their needs improved participants' morale and counteracted the sense of isolation and redundancy that often affects heart failure patients.

*You got a real feeling from the staff that they'd do anything they could for you. It was really about us and they'd go above and beyond to help you. They are great, the nurses (Centre B, R4).*

The group setting facilitated interaction with peers who had the same condition, which also lessened feelings of loneliness and anxiety.

*And then when you see other people and start talking about it, it doesn't seem nearly as bad. (Centre A, R5).*

Many of the participants had felt ill-informed about their condition prior to attending the programme. They also lacked knowledge of the tips and tricks that would have helped them cope and improve their quality of life generally. Information sessions addressed these knowledge gaps, helping them to understand their condition and manage it more successfully.

*Well, when I left hospital I was given a leaflet to read and went through them in a scattered sort of a way. It was only when I came down here that they explained it in more detail, important parts of it. (Centre A, R4).*

Just one participant had felt overwhelmed by the amount of information he received on the programme, particularly the amount of paper material, although this feeling was not shared by others.

## 5. Maintenance

A number of participants had felt somewhat directionless when the programme ended. Once they became responsible for their own regulation, it was difficult to remain motivated to maintain the health-promoting behaviours that they had developed in the clinic. Some had failed to maintain an exercise regimen, in particular the participants who had symptoms of fatigue and breathlessness. Lack of equipment was partly to blame, but the absence of the discipline of the programme was also a factor.

*I found I missed, the first week I slacked on everything because I had no place, I had no eleven o'clock to be up here and two o'clock to be up here. I kinda just went into a bit of a trough. (Centre A, R1).*

The participants were not unanimous on this point, however, and some seemed to have coped better with self-maintenance independently of the programme. These participants continued to exercise and did not feel the need for external sources of motivation.

*You have to get out and do the bit of walking or whatever, if you don't do it one day it's always harder then to do it the next. (Centre B, R2).*

## 6. Improvements

Neither group had any criticism of the programmes, although in both centres participants suggested that facilities might be improved and expanded. At present, the facilities are quite cramped as both programmes are run in a single room that accommodates both the information sessions and the exercise equipment. It was suggested that a bigger exercise room would also enable a greater number of people to benefit from each programme. 

Both groups would have welcomed a programme involving regular followup to maintain their focus and to reassure them that their self-management of the condition was appropriate and effective. Just one participant, who was “in top form”, didn't feel a need for a refresher programme on the basis that it would demand a time commitment, and that he didn't see what extra benefit it would offer.

## 7. GP Services

Participants seemed disillusioned with their GPs, which contrasted markedly with the enthusiasm they expressed for the specialist care and support from the cardiac rehabilitation staff. They generally lacked a close relationship with their GP and expressed little faith in their GP's ability to recognise and effectively meet their needs. One participant spoke of his GP's unwillingness to take responsibility in managing his condition:

*I got blood tests done … with my GP, and he got the results back and he goes …, “… I'm not taking responsibility for you, back to [rehabilitation facility]*”.* Since we finished the course, I've been back in [rehabilitation facility] six times (Centre A, R4). *

In relation to their general health problems (not specifically heart failure), many felt that GPs treated them in a summary manner, dispensing prescriptions without investigating the problem:

*You go with a pain or an ache to them and they've no test much for it, ah sure, give you an aspirin, give you Difene, give you something else for a week or a fortnight and come back. That's my main objection: there should be more serious looking into, like. (Centre A, R5).*

The GP was not seen as the first point of contact for these patients if a medical need arose. An appointment might not be available when it was needed. Even if it was, many participants were not confident that the GP could deal adequately with the situation and would simply send patients to hospital as a matter of course. If they needed advice, participants were much more likely to contact the hospital that was treating them or the cardiac unit.

*But if you have a problem at home, something went wrong with you, like what happened to me once, the last place you'd be going to would be to your GP, you'd go straight to the hospital or to the heart unit here. (Centre A, R6).*

The participants were unenthusiastic about the proposal that GPs might provide heart failure rehabilitation programmes. They felt that GP surgeries would not have the space nor the facilities to accommodate a programme. They also felt more secure doing the programme within a hospital setting:

*They know me here, they know what I've been through and what I need. He (the GP) doesn't know the half of it (Centre B, R7).*

## 8. Quality of Care

During the discussion, several participants expressed frustration at the lack of continuity in their care—for example, the doctor to whom they were referred not being available when they attended for appointments, or their regular doctor being replaced by a locum, who changed their medication, which did not work. The heart failure programmes contrasted with this experience of medical treatment. Three or four nurses ran each programme and were present consistently when participants attended. This opportunity to build a relationship with the professionals caring for them was undoubtedly an important aspect of the programme for participants. From the interactions and comments made on the day, it was clear that the patients in both groups and nursing staff with whom they worked closely shared a very good rapport.

## 9. Labelling the Condition “Heart Failure”

One participant expressed his aversion to the term “heart failure”. The term had a discouraging effect when he was first diagnosed, and he wished for an alternative. Two other participants disagreed, however, and believed it had made no difference to their response to their diagnosis:

*It's defeatist, we're failed before we start … the nurse came to me and I in the bed and I wasn't well at all … and she gave me these leaflets, Living with Heart Failure, and I said, “That's some motivation, living with heart failure”. (Centre A, R1).*

*You still have the condition, you can call it what you like, you still have the condition. To me, it doesn't matter. (Centre A, R3).*

## 10. Medication

In both hospitals a pharmacist is available as part of the multidisciplinary team to give patients advice and answer any questions that may arise. The pharmacist highlights the importance of understanding their medication, why it is prescribed and any side-effects that might arise. No one expressed having difficulty in maintaining the medication regimen. Most seemed to have accepted that their condition demanded a complex medication regimen, although some expressed exasperation with constant changes to their regimens and the slowness of new medications to take effect. Participants highlighted the need to be organised and to maintain a routine with regard to taking their medication:

*You just get used to it, you've a certain number of tablets and you just have to take them. That's the way it goes. I find the containers great [*pill containers with individual compartments*] so you know what you have to take every day and when you've already taken it. (Centre B, R5). *

One participant seemed baffled about the number of different medications he needed to take, their purpose, and their side-effects as described in the medication leaflets.

*How, God in heaven, could you be right with all them yokes, and take the leaflet out of the packet and read all the side effects, you'd go crazy. So what do you do? (Centre A, R5).*

## 11. Discussion

This study highlights the importance of formal rehabilitation for heart failure patients to enable them to cope with and adjust to the condition. Findings indicate that the programmes had met the needs of patients, and patients showed a high degree of satisfaction with the quality of care.

Taking part in a heart failure management programme was a key element in participants' recovery, helping to rebuild their confidence to resume the normal activities of daily life. Prior to participation, the fear surrounding any physical activity had had a debilitating effect on patients. In the programme environment, however, participants felt secure in being active because they were supervised by professionals. This enabled patients to negotiate and adjust to the limitations placed upon physical activity by the condition. 

Participants felt that the information sessions had imparted an understanding of the disease that they had lacked prior to attending. Knowledge of the condition is a prerequisite to self-management, and enabling patients to become expert at managing their condition is a core factor in reducing the burden of heart failure on health care services. Whilst participants in this study had received information about heart failure prior to attending the clinic, it seems that they had had difficulty in assimilating it. 

Attending the rehabilitation clinic improved considerably the morale of participants in this study. A diagnosis of heart failure causes fear and worry in patients, and many experience anxiety and depression [10]; however, maintaining morale is important to succeed in adhering to a self-care regimen [11]. Interaction with a peer group, all of whom had the same condition, enabled participants to share experiences, counteracting feelings of isolation. These patients, who had felt “written off” elsewhere in the healthcare system and had been frustrated by deficiencies in care, by contrast felt valued in the rehabilitation programme. The quality of care in the programme—the support of medical staff who were friendly and encouraging, combined with the tailoring of care to their individual needs—restored their self-esteem. 

Many heart failure patients do not undertake the recommended self-care activities, and noncompliance with medication regimens is common [12]. Participants in the current study, however, indicated that they took their medication as prescribed. This level of compliance may be due to having developed an understanding of their medication in the course of the programme. 

On the other hand, there were signs that patients neglected self-maintenance in other respects once the programme ended, in the absence of the supports of the formal care setting. In particular, some patients failed to maintain an exercise routine. This was attributed in part to lack of equipment, which suggests that patients must be supported to develop physical activity routines that are not dependent upon the equipment and structured routine of the formal rehabilitation setting. Regular, moderate daily activity is recommended for all patients with heart failure [13] and is required to sustain functional improvements made when exercise is initiated in heart failure rehabilitation [14]. Corvera-Tindel et al. [15] examined the predictors of exercise nonadherence in heart failure patients and found that patients with longer heart failure duration and increased number of comorbid conditions were less likely to adhere to a prescribed exercise regimen. Unlike patients with coronary artery disease, symptoms of depression and anxiety did not predict exercise adherence, but lower levels of hostility were found to predict nonadherence in heart failure patients. Home-based programmes using cost-effective methods that do not require specialist equipment, including walking, have shown some promise amongst heart failure patients [16]. Evangelista et al. [17] found that through home-based exercise training with advanced heart failure patients, adherence to exercise was associated with more favourable clinical outcomes. There was also a positive dose-response relationship between the amount of exercise performed and improvement in functional performance and quality of life. To facilitate adherence, clearly heart failure rehabilitation exercise programmes must take into account that exercise training and prescribed exercise must be feasible for patients in their own homes or away from the hospital environment.

Lack of motivation, however, was also a factor. A psychological support as well as a significant social outlet had been severed, leaving a number of participants feeling directionless. A need for continued external input was felt in both groups in order to maintain the health-promoting behaviours that they had started on the programme. This finding highlights that the rehabilitation needs of heart failure patients may differ from those with other cardiac conditions. Heart failure continues to place considerable self-care demands on patients even after a course of rehabilitation, and voluntary support groups for heart failure are less common than for other diseases, such as cancer. An abrupt ending of intervention may not be appropriate for many of these patients, and the provision of maintenance programmes that reduce interaction with patients gradually should be examined. 

The postrehabilitation needs of patients in this study were not being met by GPs. Patients had a low level of confidence that their GPs had sufficient expertise to manage their heart failure care. However, these misgivings may to some degree reflect the extent of tailored care provided in the rehabilitation clinic. A challenge in the future will be to develop rehabilitation or chronic disease management services for heart failure that do not undermine the role of the GP in ongoing management of patients for their heart failure and other conditions. Guidance regarding the interface of primary and secondary care is needed to manage the development of heart failure services. 

While the findings of this study are significant it is also important to note its limitations. The small size of the focus groups carried out does not allow for statistically significant generalisation of responses to the greater heart failure patient population. The fact that patients were recruited by the heart failure programme coordinator in each hospital and that participants attended the meeting voluntarily also means that the participant group may not represent views held by the wider population of heart failure patients, particularly patients that did not feel strongly about the program, those that did not complete the program and patients in poor health that may have been unable to travel to attend the focus groups. Also, because of the group nature of the focus group methodology employed, opinions presented by more assertive focus group members may have overwhelmed ideas held by other members of the group, influencing the content of the data generated. 

In conclusion, the information provided here provides some perspectives from patients living with heart failure. There is much that they are satisfied with about current service delivery. Those who were managed through a multidisciplinary chronic disease management programme tailored for heart failure were enthusiastic about its contribution to their quality of care, their quality of life and their self-care and lifestyle management. The challenge is to make such opportunities for education and support with heart failure the norm for the much wider group of heart failure patients who exist now and will grow in numbers into the future.
